# Comment on “Recent Origin and Cultural Reversion of a Hunter–Gatherer Group”

**DOI:** 10.1371/journal.pbio.0030269

**Published:** 2005-08-16

**Authors:** Tony Waters

**Affiliations:** **1**California State University at ChicoChico, CaliforniaUnited States of America

I read the article “Recent Origin and Cultural Reversion of a Hunter–Gatherer Group” [1] with interest. The article raises questions about the nature of contemporary hunter–gatherer groups like the Mlabri of Thailand that are important. But I am concerned that the authors, in demonstrating the elegance of their genetic technique, have reduced the anthropological question about socioecology to an “either–or” one of descent from an ancient isolated group versus a relatively recent “flight to the forest” by a small founder group from a horticultural society. The authors claim that genetic, linguistic, and folkloric data come down solidly on the side of the latter conclusion. I think that as likely an explanation is that the Mlabri are a product of the socioecological world of highland Southeast Asia, where most groups have varying elements of both modes of subsistence.

No Southeast Asian highlanders are strictly horticulturalists or hunter–gatherers. Most Southeast Asian highlanders are horticulturalists who supplement their diet through foraging. A few of them also trade with groups like the Mlabri, who are at one extreme of the horticulturalist–forager continuum. Sometimes, trade occurs between linguistic groups, using shared knowledge of each other's languages. Other times, trade is within the same ethnic group. Indeed, the Khmu of Laos, who are linguistically most closely related to the Mlabri, have traditionally practiced this mixed strategy.

When observed in both the 1930s by Bernatzik [2], and in the late 20th century by missionaries and anthropologists, the Mlabri were in contact with other ethnic groups, primarily the highland Hmong, Northern Thai, and Lao. Indeed, Mlabri men spoke these languages well enough to trade forest products for scraps of cloth and rice. It is also probable that, as with many other such groups, women were captured or married, and Mlabri children were occasionally taken for adoption. Checking for evidence of Mlabri mtDNA in these populations could verify whether this is the case. However, this raises a second problem with the approach the authors took. The DNA of the hill tribes presented in the article did not include those groups that the Mlabri have had contact with, such as the Hmong, northern Thai, Htin, Lao, and Khmu of the remoter areas of Nan (Thailand), Phrae (Thailand), and Sayaboury (Laos) provinces, where they have lived during at least the last 70–80 years. Instead, the authors used blood samples from different hill tribes speaking Sino-Tibetan languages and currently living in the Chiang Rai and Mae Hong Son provinces of Thailand, hundreds of kilometers to the west. These tribes have had no known contact with the Mlabri during the last 80 years, or before. In such a context, perhaps it is not surprising that the authors concluded that the Mlabri were isolated from these groups.

This opens up another explanation for how the Mlabri might have persisted in Southeast Asia during the last 600 years. They could have been skilled hunter–gatherers who 600 years ago began living in symbiotic trading relationships with more settled groups. There is no reason that such relationships could not have been persistent, even though it does not fit neatly into the old hunter–gatherer versus horticulturalist dichotomy, favored by the authors. Nevertheless, I think that this is an interesting relationship to explore. While, as the authors point out, the Mlabri may have little to teach us about how humans subsisted before the dawn of agriculture, they may well have much to say about the socioecology of how horticulturalists and hunter–gatherers coexisted since the emergence of agriculture 10,000 years ago.
